# Commentary on the effects of hypoxia on energy substrate use during exercise

**DOI:** 10.1186/s12970-019-0295-6

**Published:** 2019-07-12

**Authors:** Andrew J. Young, Lee M. Margolis, Stefan M. Pasiakos

**Affiliations:** 1 U.S. Army Research Institute of Environmental Medicine, 10 General Greene Ave, Bldg 42, Natick, MA 01760 USA; 2 Oak Ridge Institute for Science and Education, Belcamp, MD 21017 USA

**Keywords:** High altitude, Carbohydrate oxidation, Nutrition, Mountaineering

## Abstract

A recently published meta-analysis in this journal analyzed findings from studies comparing substrate use during exercise at the same relative intensity (i.e., % V̇O_2_max) in normoxic and hypoxic conditions. The primary conclusion was that hypoxia had no consistent effects on the contribution of carbohydrate oxidation to total energy expenditure. However, findings from studies comparing exercise at the same absolute intensity in normoxic as hypoxic conditions were not considered in the meta-analysis. Assessment of substrate oxidation using matched absolute intensity leads to different conclusions regarding hypoxic effects on fuel use during exercise, and that experimental model, (i.e., comparing responses to exercise at matched absolute intensity) has more practical application for developing nutritional recommendations for high-altitude sojourners. This commentary will discuss those differences.

## Background

Despite considerable research, how hypoxic exposure affects energy substrate use, particularly carbohydrate oxidation, during exercise is not fully understood. In that regard, the recent systematic review and meta-analysis reported by Griffiths et al. [1] contributes importantly toward advancing understanding of the mechanisms by which acute hypoxia^1^ affects substrate use during exercise. The primary conclusions of their analyses of 18 reported studies with a total of 170 participants were that the absolute rate of carbohydrate oxidation was lower at high altitude than at sea level, but that there were no consistent differences in percent contributions of carbohydrate and fat oxidation to total energy expenditure, when exercise responses were compared at the same relative exercise intensity (i.e., % V̇O_2_max at the altitude of the test). Further, feeding state of subjects and exercise intensity employed in the experiments accounted for a large (42%) portion of the heterogeneity in the effects of altitude on substrate use. Fed subjects and subjects exercising at higher relative intensities exhibited an increased carbohydrate oxidation in hypoxic as compared to normoxic conditions, whereas fasted subjects and subjects exercising at lower relative intensities exhibited a decrease in carbohydrate oxidation in hypoxia as compared to normoxia. Griffiths et al. [1] and others [2] attribute the lower absolute rate of carbohydrate oxidation during exercise at high altitude to the reduction in absolute intensity (power output) required to match relative intensity to that at sea level, while the similar percentage contribution of carbohydrate and fat oxidation during exercise at sea level and high altitude is attributed to the generally accepted principle that the relative mix of carbohydrate and fat oxidized during exercise is primarily regulated by relative intensity.

While Griffith et al.’s [1] approach of comparing only studies in which the relative exercise intensity was matched at sea level and high altitude provides mechanistic insights regarding regulation of substrate oxidation during exercise, the authors’ conclusion that their analyses may “inform nutritional strategies for mountaineers, military personnel and athletes during exposure to altitude, subsequently limiting the detrimental exercise performance experienced in such conditions” seems to overstate the practical application of their findings. In order to match relative intensities of exercise, the absolute exercise intensity must be lower at high altitude than sea level to offset the reduction in V̇O_2_max associated with hypoxemia at high altitude. However, since any given physical activity requires the same absolute energy (i.e., muscle power output and energy expenditure) in hypoxia as normoxia [3], findings obtained using this experimental model (matching relative exercise intensities in normoxia and hypoxia) lack practical translation to the real world. For that reason, nutritional strategies to optimize performance of mountaineers, soldiers and athletes performing at high altitude are best based on observations from experiments comparing substrate use at the same absolute exercise intensities and exergy expenditure rates in hypoxia as normoxia.

## Discussion

n contrast to comparing substrate oxidation during exercise at the same relative intensity in normoxic and hypoxic conditions, comparing exercise at the same absolute intensity generally indicates a greater contribution of carbohydrate oxidation to total energy expenditure in hypoxic than normoxic conditions (Table 1). For example, Lundby and Van Hall [2] observed that carbohydrate oxidation accounted for 74% of total energy expenditure in subjects cycling at 45% V̇O_2_max (154 W) at sea level compared to 75% (*p* > 0.05) when cycling at the same relative intensity at 4,100 m altitude. However, when these same subjects cycled at the same absolute intensity at high altitude as at sea level (154 W), carbohydrate oxidation was higher than at sea level, and accounted for 87% (*p* < 0.05) of total energy expenditure during exercise at high altitude, compared to the 75% contribution at sea level. Subsequently, Peronnet et al. [4] confirmed those findings, observing that in subjects cycling at 169 W at both sea level and 4,300 m altitude, the contribution of carbohydrate oxidation to total energy expenditure increased from 75% at sea level to 92% at altitude (*p* ≤ 0.05). The more pronounced increment in carbohydrate oxidation with hypoxia observed by Peronnet et al. [4] than Lundby and Van Hall [2], appeared due to the larger increase in relative intensity under hypoxic conditions (higher altitude, greater decrease in V̇O_2_max), which is consistent with the conclusions Griffiths et al. [1] drew from their meta-analysis.

**Table 1:** Studies comparing carbohydrate oxidation rate during exercise at the same absolute intensity at sea level and high altitude

Study	Subjects	Exercise type, intensity and VO_2_	%VO_2_max	Fed or Fasted	Altitude	Duration before test	Cho ox, g/min	Cho ox, %
Lundby & Van Hall (2002)	6 ♂ + 2♀	CE, 154 W, 60 min VO_2_ = 1.9 L/min (SL) VO_2_ = 1.9 L/min (HA)	SL = 45 HA = 54	Fed 2 h before CE	4,100 m	10 min	SL = 2.0	SL = 74
							HA = 2.5^*^	HA = 87^*^
Peronnet et al., (2006)	5 ♂	CE. 169 W, 80 min VO_2_ = 2.3 L/min (SL) VO_2_ = 2.2 L/min (HA)	SL = 54% HA = 78%	Fed, 3 h before CE	4,300 m	15 min	SL = 2.0	SL = 75
							HA = 2.6^*^	HA = 92^*^
Young et al., (2018)^a^	6 ♂	TMW, 1.6–1.7 m/sec VO_2_ = 1.6 L/min (SL) VO_2_ = 1.6 L/min (HA)	SL = 40% HA = 60%	Fasted	4,300 m	5–6 h	SL = 1.2	SL = 57
							HA = 1.1	HA = 53
Young et al., (2018)^b^	8 ♂	TMW, 1.6–1.8 m/sec VO_2_ = 1.8 L/min (SL) VO_2_ = 1.7 L/min (HA)	SL = 40% HA = 60%	Fed during TMW	4,300 m	5–6 h	SL = 1.7	SL = 78
							HA = 1.5	HA = 70

Studies measuring carbohydrate oxidation (Cho ox, g/min) and % total energy expenditure derived by Cho ox (Cho ox, %) during exercise (cycle, CE; treadmill walking, TMW) at sea level (SL) and during first 24-h at high altitude (HA). Superscripts a and b indicate different trials from same study. ^*^different from SL (*P* < 0.05); ^†^different from corresponding HA trial in that study (*P* < 0.05)

So how do these observations “inform nutritional strategies for mountaineers, military personnel and athletes during exposure to altitude?” Three reported studies have examined the impact of feeding exogenous carbohydrate on substrate oxidation during steady-state aerobic exercise at high altitude compared to at sea level [4–6]. O’Hara et al. [5] reported that total carbohydrate oxidation rate was lower at high altitude than at sea level, due to reductions in both exogenous and endogenous carbohydrate oxidation and increased fat oxidation. However, O’Hara et al. [5] compared substrate oxidation during exercise matched for relative intensity at sea level and high altitude, so the absolute exercise intensity was lower at high altitude than sea level. As discussed above, since any given physical activity requires the same absolute energy (i.e., muscle power output and energy expenditure) in hypoxia as normoxia, the findings from the experimental model used in that study [5], while informative for understanding regulatory mechanisms, do not seem to have practical application to formulating real-world nutritional guidance.

In contrast, Peronnet et al. [4], compared the effects of feeding exogenous carbohydrate on substrate oxidation during exercise matched for absolute intensity. Using this experimental model, total carbohydrate oxidation rate was higher during exercise at high altitude than sea level, but oxidation of exogenous carbohydrate was not different from sea level (although numerically, it was lower at high altitude). These findings indicate that the increased total carbohydrate oxidation was entirely supported by increased oxidation of endogenous carbohydrate stores. Similarly, our laboratory recently reported [6] that total carbohydrate oxidation rate was the same at 4,300 m as at sea level during exercise matched for absolute intensity, but oxidation rate of exogenous carbohydrate was lower at high altitude. The differences in the effect of acute hypoxia on total carbohydrate oxidation rate observed by Peronnet et al. [4] and our laboratory [6] are likely attributable to the substantially lower exercise intensity performed by participants in our study compared to that used by Peronnet et al. Collectively, it seems that during performance of a given physical task, whether aerobic exercise or prolonged strenuous work, reliance on carbohydrate oxidation for meeting energy requirements is at least the same and probably greater at high altitude than at sea level. However, feeding exogenous carbohydrate during exercise may not be as effective in sparing endogenous carbohydrate stores during exercise or work at high altitude as has been shown at sea level. Clearly, more research is needed to develop optimal carbohydrate feeding strategies for high-altitude sojourners.

## Conclusion and perspectives

In summary, the meta-analysis by Griffiths et al. [1] of studies comparing substrate metabolism during exercise at the same relative intensity in normoxic and hypoxic conditions contributes greatly to our understanding of the mechanisms by which hypoxia alters substrate metabolism during exercise. However, studies comparing substrate metabolism during exercise at the same absolute intensity in normoxic and hypoxic conditions provide the practical evidence basis for developing nutritional strategies to optimize physical performance during sojourns at high altitude. Both experimental approaches, matching relative intensity and matching absolute intensity at sea level and high altitude, are useful models. Going forward, scientists investigating effects of high altitude on energy metabolism should consider which approach most appropriately addresses their questions, or whether perhaps combining those approaches would provide an even better design.

## Data Availability

Not applicable.

## Footnotes

1 For this commentary, we only consider the effects of exposure to ≤ 24 h of hypoxia or high altitude.
